# The Temperature Dependence of Hydrogen Bonds Is More Uniform in Stable Proteins: An Analysis of NMR ^h3^J_NC′_ Couplings in Four Different Protein Structures

**DOI:** 10.3390/molecules29132950

**Published:** 2024-06-21

**Authors:** Andrei T. Alexandrescu, Aurelio J. Dregni

**Affiliations:** 1 Department of Molecular and Cell Biology, University of Connecticut, Storrs, CT 06269, USA; 2 Department of Chemistry, Massachusetts Institute of Technology, Cambridge, MA 02139, USA

**Keywords:** protein folding, *m*-value, chi-1 angle temperature dependence, structure conservation, NMR structure

## Abstract

Long-range HNCO NMR spectra for proteins show crosspeaks due to ^1^J_NC′_, ^2^J_NC′_, ^3^J_NCγ_, and ^h3^J_NC′_ couplings. The ^h3^J_NC′_ couplings are transmitted through hydrogen bonds and their sizes are correlated to hydrogen bond lengths. We collected long-range HNCO data at a series of temperatures for four protein structures. P22i and CUS-3i are six-stranded beta-barrel I-domains from phages P22 and CUS-3 that share less than 40% sequence identity. The *cis* and *trans* states of the C-terminal domain from pore-forming toxin hemolysin ΙΙ (HlyIIC) arise from the isomerization of a single G404-P405 peptide bond. For P22i and CUS-3i, hydrogen bonds detected by NMR agree with those observed in the corresponding domains from cryoEM structures of the two phages. Hydrogen bond lengths derived from the ^h3^J_NC′_ couplings, however, are poorly conserved between the distantly related CUS-3i and P22i domains and show differences even between the closely related *cis* and *trans* state structures of HlyIIC. This is consistent with hydrogen bond lengths being determined by local differences in structure rather than the overall folding topology. With increasing temperature, hydrogen bonds typically show an apparent increase in length that has been attributed to protein thermal expansion. Some hydrogen bonds are invariant with temperature, however, while others show apparent decreases in length, suggesting they become stabilized with increasing temperature. Considering the data for the three proteins in this study and previously published data for ubiquitin and GB3, lowered protein folding stability and cooperativity corresponds with a larger range of temperature responses for hydrogen bonds. This suggests a partial uncoupling of hydrogen bond energetics from global unfolding cooperativity as protein stability decreases.

## 1. Introduction

Hydrogen bonds (H-bonds) are critical components of protein secondary and tertiary structure. A main driver of protein secondary structure is that backbone H-bonds compensate for the partially polar character of the protein mainchain when it crosses through the hydrophobic core of a protein [1]. H-bonds involving sidechains and/or a solvent play additional roles in protein structure specificity, stability, and function. Individually, a H-bond confers only about 1–2 kcal/mol to the stability of the protein, the weakness of the interaction allowing it to be easily formed and broken under physiological conditions [2,3,4]. However, the tens to hundreds of H-bonds in a typical protein, together with other non-covalent interactions, provide the enthalpic stabilization of the structure that counteracts the loss of conformational entropy accompanying protein folding. The degree to which individual H-bonds contribute additively to protein stability or as part of a cooperative network of non-covalent interactions remains an open question [5,6,7]. 

Initially, H-bonds were viewed as an electrostatic interaction between a hydrogen bonded to an electronegative donor atom and a second electronegative acceptor atom [3,8]. In the 1930s, Pauling suggested that H-bonds have partial covalent character (first edition of [2]). This was finally confirmed in the late 1990s by the NMR detection of J-couplings transmitted through H-bonds [9,10,11,12,13,14] and Compton X-ray scattering anisotropy in ice [3,15]. In proteins, direct NMR investigations of H-bond couplings are usually carried out with the long-range HNCO (lrHNCO) experiment that measures a three-bond ^h3^J_NC′_ through-hydrogen-bond scalar coupling between amide ^15^N and carbonyl ^13^C’ nuclei [9]. The ^h3^J_NC′_ couplings are small with values typically <1 Hz, requiring high sensitivity and usually necessitating the perdeuteration of proteins larger than ~10 KDa [13]. However, the ^h3^J_NC′_ couplings provide an unambiguous identification of H-bond donors and acceptors, without requiring knowledge about the rest of the molecular structure. This contrasts with hydrogen exchange protection studies [16] that identify only the H-bond donor. Thus, our lab and others have found through-H-bond couplings to be extremely useful restraints in protein NMR structure calculations [17,18,19,20,21,22,23]. 

Another interesting but lesser explored application of ^h3^J_NC′_ couplings is to investigate how structure varies with conditions—a unique strength of NMR in structural biology. Arguably, ^h3^J_NC′_ couplings, because of their short distance ranges, are less complicated by averaging and dynamics than other types of NMR restraints such as RDCs, dihedral restraints, and NOEs (that can also be confounded by spin diffusion). The detection of a ^h3^J_NC′_ coupling unambiguously establishes that an H-bond between 2.5 and 3.5 Å is present, with a relatively sensitive and straightforward dependence on backbone H-bond length [24,25]. In contrast, sidechain H-bonds are seen infrequently in lrHNCO experiments, possibly due to their increased flexibility, and have a more complex dependence on H-bond length [24]. Studies on the variability of H-bonds have investigated how ^h3^J_NC′_ couplings respond to pressure [26], kosmotropic solvents like trifluoroethanol (TFE) [27], and ligand binding [28].

**Figure 1:**
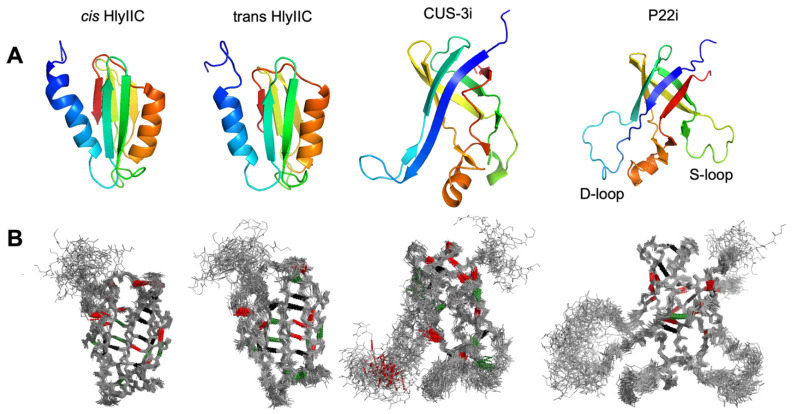
Solution structures of the four protein states studied in this work. (**A**) Cartoons of the NMR conformer closest to the ensemble average, shown with a color ramp from the N (blue) to the C-terminus (red). For HlyIIC, the largest difference between the *cis* and *trans* structures [31] is in the loop between helix α2 (orange) and strand β5 (red). The D- and S-loops in the P22i structure are dynamically disordered [32]. In Cus3i, the segment corresponding to the P22i loop becomes structured to form an extension of the β1–β2 hairpin [21]. (**B**) NMR backbone ensembles of the four proteins. Backbone H-bonds studied in this work are indicated by dotted lines. The color coding signifies changes in H-bond lengths with increasing temperature, red—increase, black—unchanged, and green—decrease. For clarity, additional depictions of H-bonds in the β-sheets of the proteins and their temperature dependencies are shown in Figures S1 and S2.

To date, there have been only two studies of the temperature dependence of ^h3^J_NC′_ couplings, for the proteins ubiquitin [29] and GB3 [30]. Both proteins showed a small weakening of ^h3^J_NC′_ couplings with increasing temperatures that was interpreted in terms of lengthening of N-to-O H-bond distances due to the thermal volume expansion of the proteins [29]. In ubiquitin, a few individual residues showed different behavior than the average H-bond expansion, characterized by H-bonds that became shorter or were invariant with temperature. These exceptions were attributed to localized partially autonomous folding regions at the ends of regular secondary structure that became more stable with increasing temperature [29]. Both the ubiquitin and G3 proteins are small (<8.5 KDa), have the same protein folding motif, and are very stable proteins (T_m_ ≥ 80 °C). We therefore wanted to investigate how generalizable the temperature dependence of H-bonds is to proteins with other structural motifs and stabilities. Since we were interested in the conservation of H-bonds between proteins with similar structures [25], we selected two pairs of structures for these studies (Figure 1). P22i and CUS-3i are autonomously folding “insertion domains” form the coat proteins of the related bacteriophages P22 and CUS-3 [21]. The two domains have 40% sequence identity and the same overall six-strand β-barrel fold, although there are structural differences in the lengths of β-strand elements and intervening loops [21]. The *cis* and *trans* states of the C-terminal domain of hemolysin II (HlyIIC) are two slightly different structural forms of the same protein sequence brought about by the *cis/trans* isomerization of the G404-P405 peptide bond. The HlyIIC domain has a pseudo-barrel fold comprised of five beta strands and two α-helices [19]. Since the *cis* and *trans* forms are in slow exchange on the NMR timescale, the two closely related structures, which differ mainly in the position of the proline-bearing loop, give separate NMR signals for about half of the residues in the protein [31]. The four protein states considered in this work were used to probe the conservation of H-bonds and their dependence on temperature in distantly and closely related protein structures.

## 2. Results and Discussion

### 2.1. lrHNCO Experiments Detect H-Bond ^3h^J_NC′_ and Sidechain ^3^J_NCγ_ Scalar Couplings

**Figure 2:**
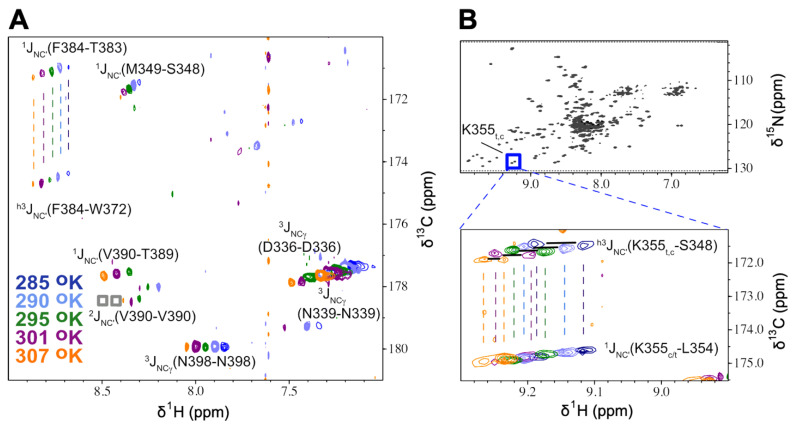
Superposed ^15^N planes from lrHNCO spectra for HlyIIC at five temperatures. (**A**) The ^15^N plane at 116.6 ppm illustrating the various types of correlations observed: ^1^J_NC′_, ^h3^J_NC′_, and ^3^J_NCγ_. Two weak intraresidue ^2^J_NC′_ crosspeaks are also present in this plane but at contour levels lower than shown (gray boxes near 8.5 ppm). (**B**) ^1^H-^15^N HSQC of HlyIIC highlighting crosspeaks due to *trans* (left crosspeaks in each pair) and *cis* (right crosspeaks in each pair) signals from residue K355. The expansion shows superpositions of 3D lrHNCO and reference HNCO planes at ^15^N 128.4 ppm for five different temperatures, using the same coloring scheme for contour levels as in (**A**). At each temperature, a pair of ^1^H_N_ resonances is observed, making it possible to investigate the temperature dependence of ^h3^J_NC′_ couplings for K355 in both the *cis* and *trans* states. There were ten residues in HlyIIC for which separate mainchain ^h3^J_NC′_ couplings could be resolved from the *cis* and *trans* states and fifteen for which NMR signals from the two conformations were unresolved (Tables S4 and S6).

Figure 2 shows representative data from lrHNCO experiments obtained at five different temperatures for the protein HlyIIC. In Figure 2A, ^15^N planes near 116.5 ppm are superposed for the different temperatures. In this portion of the spectrum, none of the six residues have resolvable signals from the *cis* and *trans* states of the protein since they are far away in the structure from the site of isomerization, P405. However, separate *cis* and *trans* signals are observed for K355 in Figure 2B. Four types of correlations are observed in the lrHNCO spectra (Figure 2A). 

The first type of crosspeak is a ^1^J_NC′_ coupling of ~11 Hz connecting the amide nitrogen with the carbonyl of the preceding residue [33], exemplified for M349, F384, and V390. These couplings are detected in standard HNCO experiments but are suppressed in the lrHNCO experiment by setting the N-to-C’ dephasing time 2T to a value of 133 ms corresponding to 2/(^1^J_NC_) [9]. For comparison, a value of ~16 ms corresponding to 1/(2 × (^1^J_NC_)) is used in the standard HNCO experiment [34]. Despite being suppressed, strong ^1^J_NC′_ couplings can nevertheless persist in the lrHNCO experiment (Figure 2). The ^1^J_NC′_ couplings are correlated with the strengths of H-bonds [33,35,36]. 

The second type of crosspeak ^h3^J_NC′_ is the three-bond through-H-bond coupling between the N atom of the H-bond donor and the C’ of the acceptor (Figure 2), which the lrHNCO experiment is intended to detect [9]. The ^h3^J_NC′_ coupling constants in units of Hz were calculated from crosspeak volumes in H-bond (V_HB_) and reference (V_ref_) versions of long-range HNCO experiments according to the formula:

^h3^J_NC′_ = ([(V_HB_/V_ref_) × (N_ref_/N_HB_)]^1/2^)/(2πT) (1)

where N_ref_ and N_HB_ are the number of scans per FID in the two experiments, and T is the 66 msec delay for the N-to-C’ INEPT refocusing period used to detect H-bond couplings [9,25]. The ^h3^J_NC′_ couplings are typically less than 1 Hz, with smaller values for H-bonds in α-helices than β-sheets [13]. It has been empirically established that the sizes of the ^h3^J_NC′_ couplings are inversely correlated with N-to-O distances across H-bonds [24,25] as

r_NO_ = 2.75 − 0.25 × (ln |^h3^J_NC′_|) (2)

assuming that the ^h3^J_NC′_ coupling constant depends only on the N-O distance [24]. 

The third type of crosspeak that occurs in lrHNCO spectra is the intraresidue two-bond ^2^J_NC′_ coupling between the N and the C’ atoms within the same residue [9,25]. These couplings are not seen in standard HNCO experiments, but their small sizes of 0 to 1.5 Hz are comparable to the ^h3^J_NC′_ couplings, so that both appear in lrHNCO experiments [9,25]. The size of the ^2^J_NC′_ coupling depends on the angle between sequentially adjacent peptide groups [37]. 

Finally, a set of relatively strong crosspeaks are seen in the lrHNCO spectra from the backbone N to the sidechain carbonyl Cγ atoms of Asp and Asn residues, for example, D336, N339, and N398 in Figure 2A. These correlations are due to ^3^J_NCγ_ couplings with sizes between 0 and 3 Hz. The ^3^J_NCγ_ couplings depend on the χ1 dihedral angles of Asp/Asn residues defined by the atoms N-Cα-Cβ-Cγ [38,39]. The ^3^J_NCγ_ coupling constant has the largest values near 3 Hz for the *trans* conformation (χ1 = 180°), decreasing to near zero for the *gauche* conformations (χ1 ± 60°). Motional averaging of the χ1 angle is expected to give ^3^J_NCγ_ values near 1.4 Hz. To characterize the ^3^J_NCγ_ couplings, we supplemented complete mainchain assignments for the four protein states with sidechain assignments for carbonyl containing Asp, Asn, Glu, and Gln using 3D HNCO and the sidechain–HCACO experiment [40]. These sidechain assignments are given in Tables S1–S3. The ^h3^J_NC′_ and ^3^J_NCγ_ coupling constants determined from lrHNCO experiments at various temperatures are summarized in Tables S4 and S5. The d(rNO)/dT slopes defining the temperature dependence of mainchain H-bond distances calculated from ^h3^J_NC′_ coupling constants are given in Tables S6 and S7, and Tables S8 and S9 compare H-bonds detected by NMR for CUS-3i and P22i with those from the cryoEM structures of the corresponding phages.

### 2.2. Agreement of H-Bonds Measured by NMR and cryoEM

None of the four protein states studied have X-ray structures; however, there are cryoEM structures for phages CUS-3 (PDB 8SKG, resolution of 2.8 Å) and P22 (PDB 5UU5 and 8I1V, resolutions of 3.3 and 2.6 Å, respectively). We compared the H-bonds detected in lrHNCO experiments in this work to the H-bonds in the I-domains of the cryoEM phage structures. 

The NMR and cryoEM data are in good agreement for CUS-3i with 31 of the 48 mainchain H-bonds (65%) seen by both NMR and cryoEM (Table S8). There were an additional 15 mainchain H-bonds in the cryoEM structure for which ^h3^J_NC′_ couplings were not detected in the lrHNCO experiment. Two of these could not be identified due to NMR spectral overlap; one is at the dynamic N-terminus of CUS-3i that is free in the NMR fragment but covalently attached to the coat protein in the cryoEM phage structure. An additional 10 H-bonds in the cryoEM structure are in turns connecting residues separated by less than three sequence positions. These are probably an artefact of overly close contacts in the cryoEM structure, since the H-bonds have marginal <DHA (donor–H•••acceptor) angles near 120° that would probably not qualify them as true H-bonds based on energetic considerations [12,13,41]. Only three genuine H-bonds disagreed between the NMR and cryoEM data. Two H-bonds, G261(N)-A259(O) and R291(N)-S303(O), were observed in the lrHNCO spectra but were not present in the cryoEM structure, with both at the end of regular β-sheet secondary structure. The Q288(N)-V273(O) H-bond in the cryoEM structure was not observed by NMR.

For P22i, the H-bonds detected in lrHNCO experiments are also consistent with those in the cryoEM structures determined at 2.6 Å-resolution [42] and an earlier 3.3 Å-resolution structure [43]. A complicating factor for comparison is that two long D- (a.a. 239–254) and S-loops (a.a. 281–291) are disordered when the P22i domain is studied in isolation by solution NMR [32], but become involved in H-bonded β-sheet structure that stabilizes the icosahedral capsid when the P22i domain is studied in the context of the intact P22 phage structure determined by cryoEM [42,43]. For the 2.6 Å-resolution cryoEM P22 structure, 25 of 37 (68%) mainchain H-bonds are detected by NMR, and 25 of 32 (78%) for the lower 3.3 Å-resolution structure. Conversely, excluding the D- and S-loops, 27 of 30 (90%) mainchain H-bonds detected by NMR for P22i are seen in one of the corresponding domains from the cryoEM structures of phage P22. As with Cus-3, the differences between the H-bonds detected by NMR and cryoEM for P22i are largely due to differences in dynamics, H-bonds that cannot be detected by NMR due to spectral overlap, and H-bonds with marginal <DHA angles in the cryoEM structures that often involve turns shorter than four residues.

By comparison, H-bonds involving sidechains show much poorer agreement between the NMR and cryoEM data. In CUS-3i, only two N-H•••O=C H-bonds involving sidechains were detected in the lrHNCO experiments and only one of these was observed in the cryoEM structure. Conversely, six of the sidechain-involving H-bonds in the cryoEM structure do not give detectable ^h3^J_NC′_ couplings. For P22i, none of the four sidechain H-bonds observed by NMR are detected in the 2.6 Å-resolution 8I1V cryoEM structure, and only one is seen in the 3.3 Å-resolution 5UU5 structure. Most of the sidechain H-bonds in the cryoEM structures are not seen in the lrHNCO NMR experiments, and there is also poor internal agreement for the sidechain H-bonds between the two cryoEM structures of P22i (Table S9). H-bonds involving sidechains are typically more difficult to detect via ^h3^J_NC′_ couplings than their backbone counterparts, possibly due to their more dynamic character [24]. In the case of P22i where multiple cryoEM structures are available, and even for high-resolution X-ray structures of the same ubiquitin protein (PDB codes 1UBQ and 2ZCC), sidechain H-bonds are poorly conserved, perhaps due to the more dynamic nature of protein sidechains. 

We next investigated the agreement of mainchain H-bond distances calculated from ^h3^J_NC′_ couplings according to Equation (2) [24] with the corresponding N-to-O distances across H-bonds in the cryoEM structures. We did not observe a correlation between distances calculated from NMR and cryoEM structures similar to those observed with X-ray structures [24,25], probably due to the more limited resolutions of the cryoEM structures. Nevertheless, when examining the RMS differences in N-to-O distances across H-bonds calculated from ^h3^J_NC′_ couplings and cryoEM, these were 0.13 Å for the 2.8 Å-resolution CUS-3 structure (31 H-bonds), 0.21 Å for the 2.6 Å-resolution P22 structure (21 H-bonds), and 0.16 Å for the 3.3 Å-resolution P22 structure (25 H-bonds). Thus, the H-bond distances from ^h3^J_NC′_ couplings agree with those from the cryoEM structures on average within about 0.1 to 0.2 Å. 

The temperature dependence of H-bonds δ(NO)/δT obtained for the proteins in this work are as large as 0.01 to 0.02 Å/°K, although for the more stable ubiquitin, the values are smaller, on average 0.0005 Å/°K (see below). To determine cryoEM structures, samples prepared at physiological temperatures are vitrified by plunging them into cryogens such as liquid ethane. Vitrification necessitates a rapid cooling rate between 10^5^ to 10^8^ °K/s^−1^ to bring samples to a typical temperature of ~80 °K for cryoEM data collection [44]. The H-bond distances obtained from ^h3^J_NC′_ couplings and cryoEM structures agree within 0.2 Å, even though the data collection temperatures for the two methods differ by more than 200 °K. This suggests that the cryoEM samples are trapped by rapid vitrification in conformations similar to those present at physiological temperatures.

### 2.3. H-Bonds and Their Temperature Dependence Are Poorly Conserved between Related Protein Structures

**Figure 3:**
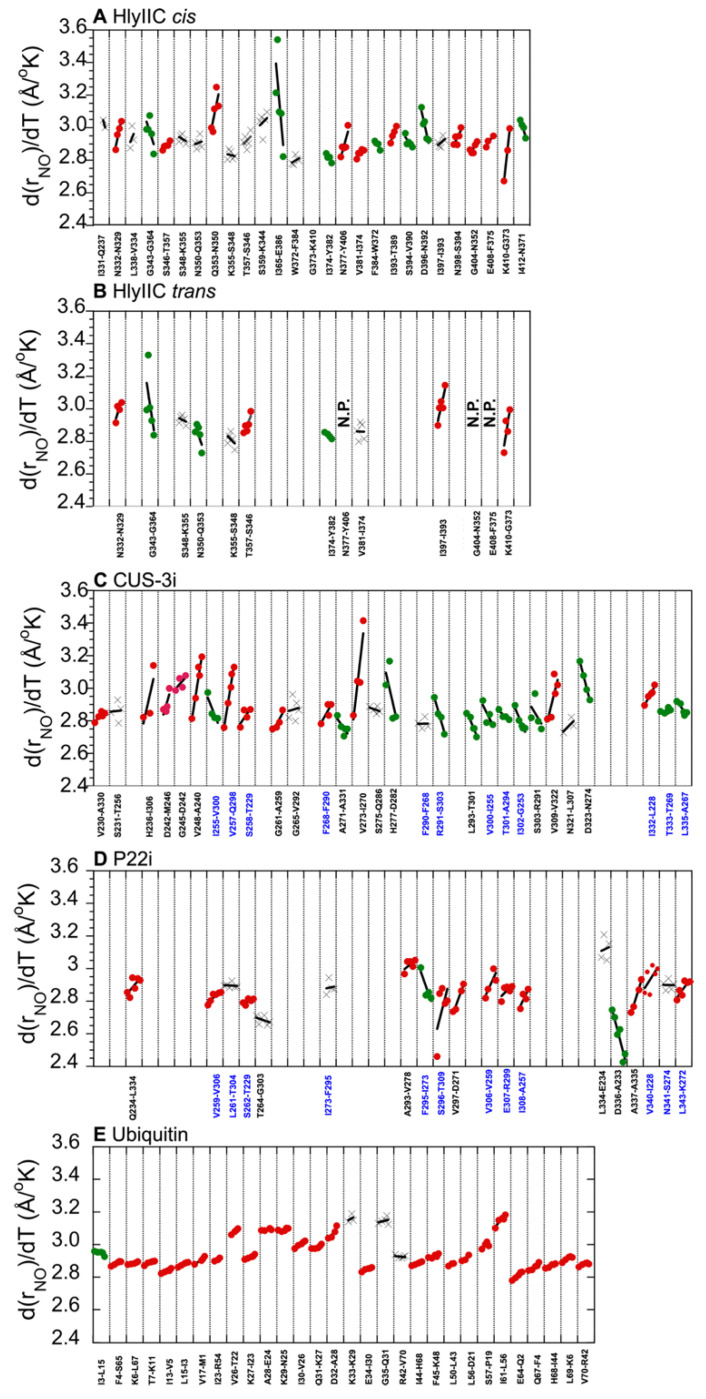
Temperature dependence of mainchain H-bond distances derived from ^h3^J_NC′_ couplings. (**A**) HlyIIC *cis* state (left to right: 285, 290, 295, 301, 307 °K), (**B**) HlyIIC *trans* state (same temperatures as *cis*), (**C**) CUS-3i (274, 286, 295, 298, 305 °K), (**D**) P22i (274, 282, 290, 298, 307, 314 °K), (**E**) ubiquitin (278, 298, 318, 328, 338 °K). The data for ubiquitin are derived from a previously published paper [29]. Each H-bond is labeled as donor (N)–acceptor (O). H-bonds that show an increase with temperature, decrease, or no change within uncertainty are shown with red, green, and gray “X” symbols, respectively. Linear fits are shown for all H-bonds but in some cases the lines are obscured by the data points. For Cus3i and P22i, H-bonds labeled as blue are structurally equivalent after the superposition of the structures. N.P. indicates three H-bonds that are present in the *cis* but not *trans* state of HlyIIC.

To obtain information on the temperature dependence of H-bonds, we recorded lrHNCO and reference HNCO spectra for the three proteins studied in this work at five or six different temperatures. ^h3^J_NC′_ coupling constants were calculated from peak volumes in the H-bond and reference spectra according to Equation (1) [9,25]. The resulting coupling constants were used to calculate N-O distances across H-bonds according to Equation (2) [24]. The derived individual H-bond distances are shown as a function of temperature in Figure 3. H-bond distances for ubiquitin, calculated the same way from published ^h3^J_NC′_ coupling constants [29], are also included for reference. The H-bond distances show three types of behaviors. Most of the distances increase with temperature, indicated by the red symbols. Some of the H-bonds become shorter and therefore stronger with increasing temperature, indicated by the green symbols. The last class of H-bonds with slopes smaller than the uncertainty of the slope and R-values < 0.6, indicated with “X” symbols, were assigned as temperature-invariant within experimental uncertainty. The proportions of different kinds of H-bond temperature responses vary among the different proteins studied (Figure 3). 

In addition to the mainchain H-bonds, we also looked at the temperature dependence of the sidechain ^3^J_NCγ_ couplings that to our knowledge has not been described before. Of 18 Asp/Asn sidechains analyzed in four protein states, 6 (33%) showed decreases in ^3^J_NCγ_ couplings with increasing temperature and the rest were invariant within experimental uncertainty (Tables S4 and S5). Most of the six residues that experienced a decrease in the coupling constant with temperature had a large sidechain ^3^J_NCγ_ above 2 Hz at low temperature, characteristic of a *trans* conformation (χ1 = 180°), that decreased towards the ~1.4 Hz limit expected for χ1 dihedrals undergoing conformational averaging [39]. 

For the analysis of mainchain H-bonds, we first looked at conservation between related structures. The phage I-domains CUS-3i and P22i share 40% sequence identity and the same six-strand β-barrel folding motif but have differences in secondary structure elements, differences in loop dynamics, and markedly different surface electrostatics [21]. The cryoEM structures of the two proteins (PDB codes 8SKG and 5UU5) align with an RMSD of 0.9 Å, allowing for the comparison of 12 structurally equivalent H-bonds after best-fit superposition. The rNO distances (Equation (2)) for equivalent H-bonds show only a moderate correlation between the two proteins (R-value = 0.76, *p* = 0.0038), which could be due to the proteins sharing similar secondary structures and the fact that H-bonds are shorter in β-sheets. The d(rNO)/dT slopes describing the changes in H-bond distances with temperature were not significantly correlated between the two proteins (R-value = −0.50, *p* = 0.093). 

We next examined the *cis* and *trans* states of HlyIIC related by isomerization about the G404-P405 peptide bond. The two states are in slow exchange on the NMR timescale, giving separate NMR signals for about half of the residues in the protein [45]. The main difference between the structures of the two states is in the orientation of the loop between helix α2 and strand β5 that harbors P405 [31]. We were able to resolve and analyze ten structurally equivalent H-bonds that were resolved in the *cis* and *trans* states. The H-bond distances at 307 °K are moderately correlated (R-value = 0.69, *p* = 0.026). However, in the same 3D lrHNCO spectrum where the *cis* and *trans* state can be analyzed simultaneously, 3 of 28 H-bonds in the *cis* state are not seen in the *trans* state (N377-Y406, G404-N352, E408-F375). The three H-bonds involve residues near the P405 site of isomerization that are lost in the *trans* state due to the structural differences accompanying isomerization. The d(rNO)/dT slopes for the ten structurally equivalent H-bonds are only moderately correlated between the *cis* and *trans* states (R-value = 0.80, *p* = 0.0059). In several cases, N350-Q353, T357-S346, V381-I374, and I397-I393, the H-bonds show markedly different d(rNO)/dT slopes between the two states.

The relatively weak conservation of H-bond distances and temperature responses between closely similar protein structures such as the *cis* and *trans* states of HlyIIC suggest that the length and temperature dependence of H-bonds are determined mostly by short-range interactions in their immediate vicinities and less by the overall protein fold. This is further supported by the observation that three H-bonds are lost near P405 in the *trans* compared to the *cis* state of HlyIIC due to conformational differences localized to the loop bearing the proline. 

### 2.4. The Variability of H-Bond Temperature Responses Is Inversely Correlated with Global Folding Stability 

**Figure 4:**
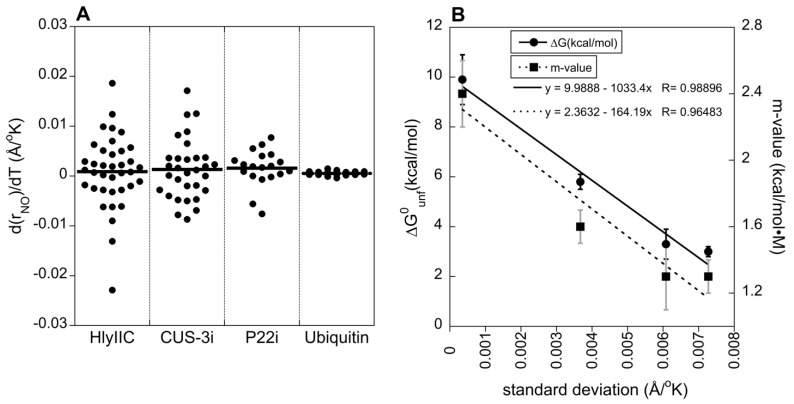
Variability in the temperature dependence of H-bonds as a function of folding stability. (**A**) The distribution of d(r_NO_)/dT slopes in four proteins arranged in order of increasing stability to unfolding (least stable on right, most stable on left). The data for HlyIIC include H-bond data for both the *cis* and *trans* states, which have very similar unfolding stabilities [31]. (**B**) Correlation between the standard deviation in d(r_NO_)/dT slopes and parameters related to protein stability: ∆G^0^_unf_ and *m*-values (from Table 1).

Data on the stabilities of the three proteins used to carry out temperature-dependent H-bond studies in this work, together with ubiquitin from a previously published study [29], are given in Table 1. A plot summarizing the variability in d(rNO)/dT slopes is shown in Figure 4A. The proteins in Figure 4A are arranged in order of increasing stability to unfolding from left to right (Table 1). The average d(rNO)/dT slope near (10.7 ± 4.6) × 10^−4^ Å/K is similar for all the proteins. We used the *t*-test to establish that the difference in the means of the d(rNO)/dT slopes is not statistically significant (*p* > 0.05) with any pairing of the proteins. The average d(rNO)/dT slope of (10.7 ± 4.6) × 10^−4^ Å/K probably reflects the thermal volume expansion coefficient, an intrinsic property that has a conserved value near 5.2 × 10^−4^ 1/K for a variety of proteins [29]. The spread in d(rNO)/dT slopes, however, shows an increase with decreasing stability to unfolding. For the moderately stable protein HlyIIC, the range of d(rNO)/dT slopes between 0.019 and −0.022 Å/K is more than 10-fold larger than for the stable protein ubiquitin where d(rNO)/dT slopes vary between 0.0015 and −0.0004 Å/K. This is illustrated in Figure 4B where the standard deviation of the d(rNO)/dT slopes is correlated with the *m*-values and ∆G^0^_unf_ values obtained for the three proteins from equilibrium unfolding experiments (Table 1). Studies of the temperature dependence of H-bond ^h3^J_NC_ couplings were performed for the additional protein GB3 [30] but are not included in Figure 4 because data for the couplings of individual H-bonds were not available. Nevertheless, the range of d(^h3^J_NC_)/dT slopes between 0.001 and −0.0003 Hz/K for GB3 [30] is very similar to those for ubiquitin [29]. The GB3 protein has a similar fold to ubiquitin [46], and a high thermal stability estimated to be ≥90 °C [47,48]. Therefore, the data for GB3 also qualitatively support that d(rNO)/dT slopes are more uniform for stable proteins. 

The *m*-value is a descriptor of the slope or steepness of the unfolding transition [53,54,55]. There are two interpretations of the *m*-value. The first is that it describes the change in solvent accessible surface area (∆ASA) between the folded and unfolded state [53,54]. The second interpretation, which we favor to explain the variance in d(rNO)/dT H-bond slopes, is that the *m*-value describes the cooperativity of the unfolding transition [55,56]. The two interpretations are largely equivalent, in as much as cooperative all-or-none unfolding transitions lead to a large change in accessible surface area whereas non-cooperative partial unfolding leads to a smaller change in accessible surface area. The variance in d(rNO)/dT H-bond slopes is also correlated with ∆G^0^_unf_, the change in the Gibbs free energy for unfolding measured in equilibrium denaturation experiments that describes the stability of the folded compared to the unfolded state. It is well established that ∆G^0^_unf_ and the *m*-value for unfolding are often correlated [50,54]. The ∆G^0^_unf_ and *m*-values are also correlated for the four proteins considered in Table 1 and Figure 4 (R-value = 0.991, *p* = 0.0088). The *m*-values tend to be small for poorly structured proteins such as molten globules, and large for stable proteins [55,57]. 

The correlation between increased variability in H-bond d(rNO)/dT slopes and lower *m*-values (Figure 4B) is consistent with the interpretation of the latter in terms of cooperativity. For the highly cooperative and stable protein ubiquitin, all H-bonds show nearly the same small 5 × 10^−4^ Å/K expansion that coincides with the value for the thermal volume expansion of the protein. For the least stable proteins, CUS-3 and HlyIIC, with the smallest *m*-values indicative of diminished unfolding cooperativity, the range of d(rNO)/dT slopes is about 10-fold larger and includes an increasing proportion of H-bonds with negative slopes, shown in green in Figure 3. Most of the H-bonds with negative d(rNO)/dT slopes occur in irregular structures, or at the edges of secondary structure elements. In the HlyIIC β-sheet, H-bonds with negative d(rNO)/dT slopes are segregated largely to strands β5–β3–β4 while strands β1–β2 have H-bonds that are invariant or have positive slopes (Figure S1). Based on hydrogen exchange data, the two sheets in HlyIIC have properties of independent folding subdomains, with the β5-β3-β4 sheet being more stable to exchange than β1–β2 [19]. In the β-barrel of CUS-3i, the H-bonds with negative d(rNO)/dT slopes are segregated to specific β-strand pairings between strands β2–β4–β5 and between strands β3–β6 (Figure S2). The segment β2–β4–β5 in CUS-3i behaves as an autonomously folding subdomain under acid denaturing conditions, retaining β-sheet structure while the rest of the protein is unfolded (A.T.A, unpublished observations). Taken together, these observations suggest that H-bonds with negative d(rNO)/dT slopes occur in protein subdomains that have enhanced stability to unfolding. 

Residues with negative d(rNO)/dT slopes tend to correspond to some of the shortest H-bonds in the proteins HlyIIC, CUS-3i, and P22i (green in Figure 3). This suggests that the H-bonds are energetically favorable but opposed by strain, arising from the remainder of non-covalent interactions stabilizing the structure. In weekly cooperative proteins, as the structure becomes increasingly dynamic at higher temperature, the strain due to rigid sidechain packing could become abated, allowing some of the H-bonds to move towards their energetic optimum. In contrast, in a highly cooperative stable system like ubiquitin, nearly all H-bonds experience the same increase driven by thermal expansion, with the few exceptions of H-bonds in the β-strand near the N-terminus that are invariant or become more stable at higher temperature [29]. 

The H-bonds may not be undergoing changes in length with temperature but changes in the ratio of conformers that have the H-bonds formed or broken. These conformers are in fast exchange on the NMR timescale, so that the measured ^h3^J_NC′_ coupling will be a population-weighted average. When the α-helical structure of the RNaseA S-peptide is stabilized by increasing concentrations of the kosmotropic solvent TFE, the ^h3^J_NC′_ coupling constants show apparent increases suggestive of decreasing H-bond distances [27]. However, circular dichroism (CD) ellipticity becomes more negative [58] and the NMR Cα secondary chemical shifts increase [27]. Both observations are more consistent with an increasing fraction of molecules adopting H-bonded α-helical conformers than with a shortening of pre-existing H-bonds. Similarly, in proteins with low *m*-values indicative of reduced unfolding cooperativity, the responses of H-bonds to changes in temperature would be more varied if the populations of individual sub-structures become uncoupled from the unfolding of the overall global structure. Our conclusions for this work are tempered by the fact that the number of proteins for which data are available is currently small. Future studies on the temperature dependence of H-bonds as well as other NMR variables such as mainchain *S*^2^-order parameters [59,60] should clarify how temperature effects depend on the cooperativity of protein structure. 

## 3. Materials and Methods

### 3.1. Samples

Double-labeled ^13^C/^15^N and triple-labeled ^13^C/^15^N/^2^H samples of wild-type HlyIIC [19], P22i [61], and CUS-3i [62] were prepared as described previously. For long-range HNCO experiments to measure H-bonds, triple-labeled ^13^C/^15^N/^2^H proteins in 90% H_2_O/10% D_2_O were used with the following sample conditions: 1.0 mM HlyIIC in 20 mM NaH_2_PO_4_ with 1 mM EDTA and 1 mM PMSF at pH 6.6; 1.7 mM P22i in 20 mM NaH_2_PO_4_ at pH 6.1; and 1.6 mM CUS-3i in 20 mM NaH_2_PO_4_ at pH 6.3. For sidechain–HCACO experiments to obtain Asp/Asn and Glu/Gln assignments, double-labeled ^13^C/^15^N samples were lyophilized and re-dissolved in 99.96% D_2_O with the following sample conditions: 1 mM HlyIIC at pH* 6.0, 3.8 mM P22i at pH* 6.1, and 0.7 mM CUS-3i at pH* 6.3, where pH* is the pH measured in D_2_O with a glass pH electrode.

### 3.2. NMR Data Acquisition and Analysis

Experiments were performed on 600 MHz Bruker Avance and 600 MHz Varian Inova spectrometers, both equipped with cryogenic probes. For the Bruker instrument (Bruker, Billerica, MA, USA), we modified the BEST TROSY HNCO H-bond experiment [63] from the Bruker IBS library (pulse sequence *BT_HNCO_hbonds*) to work on Topsin v. 2.1. At each temperature, we obtained long-range HNCO (d23 = 66 ms) and reference HNCO (d24 = 16.5 ms) versions of the experiments to quantify ^h3^J_NC′_ couplings [9,25,27]. For HlyIIC, we recorded 3D spectra on the Bruker 600 MHz spectrometer at temperatures of 285, 290, 295, 301, and 307 °K, with 32(t1,C’) × 16(t2,N) × 1024(t3,H) complex points and acquisition times of 13, 9, and 114 ms in the t1, t2, and t3 dimensions. Total experiment times were on the order of 46 and 4 h for the H-bond and reference spectra, respectively. For CUS-3i, we recorded 3D spectra on the Bruker 600 MHz spectrometer at temperatures of 274, 286, 295, 298, and 305 °K, with 64(t1,C) × 16(t2,N) × 1024(t3,H) complex points and acquisition times of 26, 10, and 107 ms in the t1, t2, and t3 dimensions. Total experiment times were about 54 and 8 h for each of the H-bond and reference spectra, respectively. Since P22i had the best dispersion of the three proteins, we recorded 2D versions of the TROSY-HNCO experiment (pulse sequence *best_trosy_hbonds*) on the Varian 600 MHz spectrometer (Palo Alto, CA, USA) at temperatures of 274, 282, 290, 298, 307, and 314 °K. For the reference experiment, we modified the Varian *best_trosy_hbonds* pulse sequence to shift the ^13^C′ 180° pulses by 16.5 ms with respect to the ^15^N 180° pulses in the INEPT steps as described in the literature [9]. The 2D data sets on P22i were recorded with 64 (t1,C) × 512 (t2,H) complex points with acquisition times of 14 (t1) and 107 ms (t2). Total acquisition times were 11 h for the H-bond and 1 h for the reference experiments. NMR sample temperatures were calibrated using 100% methanol (T < 300 °K) and 100% ethylene glycol (T ≥ 300 °K) standards, as described in the Bruker VT-calibration manual.

Sidechain carbonyl and amide resonances were assigned from 3D HNCO and HCACO experiments. The 3D HCACO experiment (Bruker pulse sequence *hcacogp3d*) was modified for sidechains as described in the literature [40]; namely, the aliphatic ^13^C center was placed at ~39 ppm and the carbonyl ^13^C center at ~177 ppm, and the delay 1/[4(J_HC_)] (called d4 in the *hcacogp3d* pulse sequence) was set to 1.8 ms for methylene protons rather than 3.3 ms in the standard experiment. The 3D HCACO spectrum for HlyIIC was recorded at a sample temperature of 37 °C on a 500 MHz instrument with 50(t1,C) × 32(t2,C′) × 1024(t3,H) complex points, and acquisition times of 20, 12, and 122 ms in the t1, t2, and t3 dimensions, respectively. The total acquisition time was 34 h. The 3D HCACO spectrum for CUS-3i was recorded at a temperature of 25 °C on a 500 MHz Bruker magnet with 16(t1,C) × 11(t2,C′) × 1024(t3,H) complex points, and acquisition times of 4 (t1), 3 (t2), and 157 (t3) ms; the total was 30 h. The 3D HCACO for P22i was at 37 °C on a 600 Bruker MHz spectrometer with 48(t1,C) × 32(t2,C′) × 1024(t3,H) complex points, and acquisition times of 29 (t1), 13 (t2), and 122 (t3) ms; the total was 64 h.

NMR spectra were processed with iNMR v. 6.3 (Mestrelab Research, Santiago de Compostela, Spain) and analyzed with CcpNmr Analysis v. 2.5.2 [64]. Published assignments were used to analyze the NMR data for *cis* HlyIIC (BMRB 19461), *trans* HlyIIC (BMRB 19462), CUS-3i (BMRB 25263), and P22i (BMRB 18566). 

^h3^J_NC′_ coupling constants were calculated according to Equation (1). To estimate uncertainties in ^h3^J_NC′_ values, experiments were replicated on two separate samples at one temperature for each protein: HlyIIC (307 °K), CUS-3i (298 °K), and P22i (307 °K). The RMS differences between the duplicate ^h3^J_NC′_ values were 0.10, 0.06, and 0.04 Hz for HlyIIC, CUS-3i, and P22i, respectively. H-bond distances were calculated using the empirical relationship (Equation (2)) established by Bax and co-workers [24]. 

### 3.3. Structure Analysis

To correlate NMR with structural parameters, we used the segment corresponding to CUS-3i (chain A of the asymmetric unit, residues 223–337) from the 2.8 Å-resolution cryoEM structure of phage CUS-3 (Richard Whitehead, Carolyn Teschke, and A.T.A, in preparation; PDB accession code 8SKG, coordinates are available from the corresponding author until release). For P22i, we used the 3.3 Å-resolution cryoEM structure of phage P22 (PDB 5UU5, chain E of asymmetric unit, residues 223–345 [43]), and the 2.6 Å-resolution cryoEM structure of phage P22 (PDB 8I1V, chain A of asymmetric unit, residues 223–345 [42]). Interatomic distances were calculated with the “get_distance” command of PyMol v. 2.5.5 (Schrödinger software, New York, NY, USA). H-bonds were calculated with the program HBPLUS v. 3.06 [65].

## Figures and Tables

**Table 1:** Folding stability information on proteins used for lrHNCO studies ^a^.

Protein	∆G^0^_unf_ (kcal/mol)	m-Value (kcal/mol·M)	T_melt_ (°C)	References
HlyIIC ^b^	3.0 ± 0.2	1.3 ± 0.1	52	[19,31]
CUS-3i	3.3 ± 0.6	1.3 ± 0.2	48	[49]
P22	5.8 ± 0.3	1.6 ± 0.1	54	[50]
ubiquitin	9.9 ± 1.0	2.4 ± 0.2	>90	[51,52]
GB3	~6 to 7	N.D.	≥90	[47,48]

^a^ The ∆G^0^_unf_ and *m*-values are from equilibrium denaturation experiments using urea, except for ubiquitin where guanidine chloride was used as a denaturant, and GB3 where values were estimated from the highly homologous GB1 and GB2 domains [47,48]. The T_melt_ values are midpoints for thermal unfolding. ^b^ Urea and temperature denaturation data for HlyIIC were obtained by CD spectroscopy, which does not distinguish between the *cis* and *trans* states. As such, the reported values are population-weighted averages for the two states, which have very similar stabilities to unfolding. At the 298 K temperature used for the urea denaturation studies, the *trans*/*cis* ratio is 1:1 so the ∆∆G*_trans_*_->*cis*_ ~ 0 kcal/mol. At higher temperatures, the *trans* state becomes slightly favored, with the *trans*/*cis* ratio reaching 1.5:1 at a temperature of 310 K [31].

## Data Availability

The data presented in this study are available in article and Supplementary Materials.

## Supplementary Materials

The following supporting information can be downloaded at: https://www.mdpi.com/article/10.3390/molecules29132950/s1. Tables S1–S3: sidechain carbonyl NMR assignments; Tables S4 and S5: ^3h^J_NC′_ and ^3^J_NCγ_ couplings at various temperatures; Tables S6 and S7: temperature dependences of H-bond lengths; Tables S8 and S9: comparisons between NMR and cryoEM H-bonds in CUS-3i and P22i. Figures S1 and S2: schematics illustrating H-bonds and their temperature dependence in the β-sheets of HlyIIC, CUS-3i and P22i.

## Abbreviations

2D and 3D, two- and three-dimensional; cryoEM, cryogenic electron microscopy; CUS-3i, insertion domain from the coat protein of phage CUS-3; FID, free induction decay; H-bond, hydrogen bond; HlyIIC, hemolysin II C-terminal domain; INEPT, insensitive nuclei enhancement by polarization transfer; lrHNCO, long-range HNCO experiment; NMR, nuclear magnetic resonance; P22i, insertion domain from the coat protein of phage P22; RMS, root-mean-square; RMSD, root-mean-square deviation; TFE, trifluoroethanol; TROSY, transverse relaxation optimized spectroscopy.
