# Supplementary material for: The Temperature Dependence of Hydrogen Bonds Is More Uniform in Stable Proteins: An Analysis of NMR h3JNC′ Couplings in Four Different Protein Structures
Source: Molecules. 2024 Jun 21;29(13):2950. doi: 10.3390/molecules29132950 (PMC11243222; doi:10.3390/molecules29132950)
Supplement: Supplementary file 1 [file molecules-29-02950-s001.zip › molecules-3040391-supplementary.pdf]

Supplementary Information for

The temperature dependence of hydrogen bonds is more uniform in stable proteins: analysis of NMR $^{h3}J_{NC'}$ couplings in four different protein structures

Andrei T. Alexandrescu[1*] and Aurelio J. Dregni[2]

[1]*Department of Molecular and Cell Biology, University of Connecticut, Storrs, CT 06269, USA*

[2]*Department of Chemistry, Massachusetts Institute of Technology, Cambridge, MA 02139, USA*

Table of contents:

Table S1 – HlyIIC sidechain carbonyl assignments
Table S2 – P22i sidechain carbonyl assignments
Table S3 – CUS-3i sidechain carbonyl assignments
Table S4 – ^{3h}JNC' and ^{3}JNCγ couplings (Hz) at various temperatures for HlyIIC
Table S5 – ^{3h}JNC' and ^{3}JNCγ couplings (Hz) in CUS-3i and P22i at various temperatures
Table S6 – Temperature dependence of H-bonds in HlyIIC
Table S7 – Temperature dependence of H-bonds in CUS-3i and P22i
Table S8 – Comparison between CUS-3i NMR and cryoEM H-bonds.
Table S9 – Comparison between P22i NMR and cryoEM H-bonds.

Figure S1 – Temperature dependence of β-sheet H-bonds in *cis* and *trans* HlyIIC
Figure S2 – Temperature dependence of β-sheet H-bonds in CUS-3i and P22i

Table S1. HlyIIC sidechain carbonyl assignments[a]

Major *cis* chemical shifts (ppm)

	Hβ/γ[b]	Cβ/Cγ[b]	C'	N	HN
Glu 325 (7)[c]	2.46, 2.33	36.89	183.66[d]		
Glu 326 (8)	2.31, 2.17	36.33	183.82[d]		
Glu 368 (50)	2.30, 2.18	37.17	184.23[d]		
Glu 379 (61)	2.25	34.36	180.21		
Glu 386 (68)	2.93, 2.29	39.42	181.09		
Glu 408 (90)	2.20, 2.12	37.45	183.43		
Asp 319 (1)	2.89, 2.83	39.14	177.02		
Asp 336 (18)	2.72	41.67	177.90		
Asp 369 (51)	2.95, 2.44	39.70	180.58		
Asp 396 (78)	3.11, 2.27	39.70	179.62		
Gln 320 (3)	2.44, 2.35	34.42	179.84	110.67	7.49,7.27
Gln 327 (9)	2.38	34.36	180.45		
Gln 353 (35)	2.31, 2.18	36.05	183.80[d]	111.58	7.37,6.63
Gln 411 (93)	1.05	33.80	180.75	110.68	7.02,6.66
Asn 320 (2)	2.89	41.67	175.20	109.78	7.99,7.47
Asn 329 (11)	2.93, 2.87	38.63	176.98	112.03	7.74,7.00
Asn 332 (14)	3.00	38.07	176.97	111.36	7.44,6.91
Asn 335 (17)	3.08, 2.86	38.30	175.24	109.76	7.92,7.47
Asn 339 (21)	3.01, 2.60	38.01	177.61	110.68	7.71,6.90
Asn 350 (32)	2.77, 2.55	39.42	176.95	112.50	7.62,6.93
Asn 352 (34)	3.07, 2.97	39.14	178.09	111.14	7.09,6.78
Asn 360 (42)	3.19, 3.06	37.17	178.07	112.03	7.78,6.96
Asn 371 (53)	2.56, 2.10	42.80	176.63	116.17	7.59,7.25
Asn 377 (59)	3.08	37.73	178.82	109.31	7.36,7.04
Asn 385 (67)	2.75, 2.67	43.92	176.54	113.45	7.41,7.41
Asn 392 (74)	2.99	38.30	176.04	112.50	7.77,7.07
Asn 395 (77)	2.95, 2.87	38.86	175.43	112.50	7.81,6.86
Asn 398 (80)	2.78, 2.67	41.11	179.91	113.00	7.85,7.23

Asn 401 (83)	3.01, 2.60	37.45	177.82	110.22	7.37,6.63
**Minor *trans*** chemical shifts (ppm)					
Asp 319 (1)[e]	2.88, 2.78	39.38	175.85		
Asp 336 (18)	2.82, 2.70	41.67	176.56		
Asp 369 (51)	2.94, 2.44	40.26	180.56		
Asp 396 (78)	2.92, 2.29	39.42	181.07		
Gln 353 (35)			177.63	111.12	7.62,6.70
Asn 329 (11)	2.86, 2.82	38.58	175.75		
Asn 339 (21)	3.01, 2.59	36.61	177.78	110.68	7.62,6.86
Asn 352 (34)	2.80, 2.74	39.14	176.95	112.50	7.62,6.99
Asn 371 (53)	2.72	41.95	178.81	116.17	8.09,7.21
Asn 392 (74)	2.87, 2.81	39.98	177.01	112.50	7.61,6.93
Asn 398 (80)	2.87, 2.80	39.09	178.36	113.00	7.82,7.23

[a]Sample conditions: pH* 6.0 in D_2O and pH 6.6 in H_2O, both at a temperature of 37 °C.

[b]Hβ and Cβ shifts are listed for Asp/Asn, and Hγ and Cγ are listed for Glu/Gln.

[c]The first number is the residue position in the full-length HlyII protein. The second number in parentheses is the residue position in the HlyIIC domain, to match the numbering schemes of the BMRB entries 19461, 19462, and 19463.

[d]Corrected chemical shift for a C' resonance aliased outside of the ^{13}C' spectral window.

[e]The assignments for this residue are tentative

Table S2. P22i sidechain carbonyl assignments[a]

chemical shifts (ppm)

	Hβ/γ[b]	Cβ/Cγ[b]	C'	N	HN
Glu 307	2.05, 2.01	36.76	182.02		
Glu 323	2.44, 2.33	37.10	184.19		
**Asp 246[c]**	2.79, 2.64	41.76	180.37		
Asp 253	2.88, 2.60	41.82	180.35		
Asp 271	2.76, 2.55	41.48	178.89		
Asp 292	2.91, 2.43	41.01	179.14		
Asp 302	3.22, 3.20	39.33	180.77		
Asp 316	3.15, 2.63	40.11	180.77		
Asp 317	3.34, 2.65	40.78	179.94		
Asp 336	2.52, 2.48	42.06	179.06		
Gln 234	2.23, 2.04	34.39	180.34[d]	111.13	7.41,6.80
**Gln 242**	2.49, 2.43	30.90	179.65	113.78	7.62,7.00
**Gln 283**	2.48, 2.46	34.61	180.09[d]	112.19	7.60,6.93
**Gln 291**	2.46, 2.45	33.90	180.77[d]	112.19	7.57,6.86
Gln 324	2.60, 2.31	34.66	179.23	110.86	7.59,6.86
**Asn 245**	2.83, 2.75	38.96	176.98	112.72	7.70,7.01
**Asn 248**	2.86, 2.70	39.11	176.99	112.72	7.71,7.04
**Asn 251**	2.74, 2.62	39.01	176.90	112.72	7.60,6.89
**Asn 254**	2.62, 2.52	39.43	176.81	111.66	7.42,6.85
**Asn 287**	2.84	39.00	177.60	112.19	7.87,7.21
Asn 329	3.26, 3.03	39.38	177.25	106.35	7.87,7.21
Asn 331	2.99, 2.84	36.70	177.03	114.21	8.12,7.10
Asn 341	2.61, 2.52	41.20	176.82	112.59	7.42,6.74
**Asn 344**	2.80, 2.74	39.09	177.17	112.72	7.61,6.93

----

[a]Sample conditions: pH 6.1 and a temperature of 37 °C.
[b]Hβ and Cβ shifts are listed for Asp/Asn, and Hγ and Cγ are listed for Glu/Gln.
[c]Residues in bold type are in the dynamically disordered regions of P22i.
[d]Corrected chemical shift for a C' resonance that were aliased outside of the ^{13}C' spectral window.

Table S3. CUS-3i sidechain carbonyl assignments[a]
chemical shifts (ppm)

	Hβ/γ[b]	Cβ/Cγ[b]	CO	N	HN
Glu 226 (7)	2.32, 2.34	35.56	180.89		
Glu 235 (16)	2.22, 2.24	34.61	180.73		
Glu 239 (20)	2.18, 2.19	36.25	180.44		
Glu 310 (91)	2.29, 2.31	36.01	180.84		
Glu 328 (109)	2.39, 2.43	35.56	178.15		
Asp 242 (23)	3.24, 3.16	40.95	177.55		
Asp 249 (30)	2.75, 2.81	40.61	176.04		
Asp 266 (47)	2.53, 2.97	41.41	176.77		
Asp 282 (63)	2.57	40.68	177.46		
Asp 313 (94)	2.67, 2.44	41.87	177.53		
Asp 323 (104)	2.89, 2.78	38.86	176.06		
Gln 233 (14)	2.06	34.12	176.02	113.17	6.69,6.86
Gln 252 (33)	2.11, 2.14	33.03	177.85	110.54	6.62,7.43
Gln 263 (44)	2.34, 2.38	33.22	175.96	112.24	6.79,7.60
Gln 278 (59)	2.50	33.30	178.32	111.63	6.82,7.61
Gln 286 (67)	2.32	32.44	178.65	112.27	6.84,7.48
Gln 288 (69)	1.77,1.87	32.40	180.28	112.08	6.95,7.93
Asn 244 (25)	2.88	38.92	175.48	114.29	6.93,7.82
Asn 250 (31)	2.89	38.43		116.30	7.75,7.40
Asn 274 (55)	2.67	39.73	177.73	112.91	6.51,7.85
Asn 311 (92)	2.89, 2.91	37.67	175.28	112.56	7.09,7.69
Asn 321 (102)	2.78, 2.80	39.12	173.76	107.10	7.26,8.11
Asn 336 (117)	2.90	38.24	174.61	114.74	8.56,8.18

[a]Sample conditions: pH 6.3 and a temperature of 25 °C.

[b]Hβ and Cβ shifts are listed for Asp/Asn, and Hγ and Cγ are listed for Glu/Gln.

Table S4. **^{3h}JNC' and ^{3}JNCγ couplings (Hz) at various temperatures for HlyIIC**[a]

			Major cis form or unresolved					Minor trans form				
**Don.**	**Acc.**	**2°**	**285°K**	**290°K**	**295°K**	**301°K**	**307°K**	**285°K**	**290°K**	**295°K**	**301°K**	**307°K**
I331	Q327	α1				0.37	0.31					
N332	N329	α1		0.63	0.44	0.38	0.31		0.52	0.35	0.38	0.31
L338	V334	α1	0.61	0.35	0.50							
G343	G364	turn	0.39	0.38	0.27	0.43	0.70	0.38	0.10	0.36	0.49	0.70
S346	T357	β1	0.64	0.58	0.58	0.57	0.51					
S348	K355	β1	0.52	0.45	0.43	0.50	0.55	0.52	0.45	0.43	0.66	0.56
N350	Q353	β1	0.53	0.62	0.52	0.43	0.59	0.65	0.54	0.58	0.70	1.09
Q353	N350	turn	0.37	0.41	0.23	0.14	0.22					
K355	S348	β2	0.81	0.62	0.67	0.79	0.74	0.86	0.72	0.64	0.77	1.01
T357	S346	β2	0.53	0.46	0.63	0.76	0.39	0.66	0.56	0.63	0.54	0.39
S359	K344	β2	0.31	0.28	0.50	0.30	0.25					
I365	E386	irreg	0.16	0.04	0.25	0.26	0.75					
I365	K387	irreg	0.98	0.88	1.16	1.02	0.85					
W372	F384	β3	0.89	0.92	0.72	0.79	0.83					
G373	K410	β3	0.94	0.98	0.87	0.75	0.84					
I374	Y382	β3		0.70	0.77	0.76	0.87	0.66		0.69	0.73	0.77
N377	Y406	irreg	0.75	0.59	0.61	0.59	0.35	NP[b]	NP	NP	NP	NP
V381	I374	β4	0.80	0.69	0.69	0.63	0.64	0.63	0.82	0.51	0.55	0.77
F384	W372	β4		0.52	0.54	0.55	0.64					
I393	T389	α2		0.54	0.45	0.41	0.36					
S394	V390	α2	0.42	0.55	0.53	0.56	0.59					
D396	N392	α2	0.22	0.34	0.32	0.48	0.50					
I397	I393	α2	0.53	0.58	0.60	0.44	0.51	0.21	0.36	0.31	0.36	0.55
N398	S394	α2	0.56	0.46	0.56	0.42	0.37					
G404	N352	irreg	0.64	0.68	0.68	0.56	0.52	NP	NP	NP	NP	NP
E408	F375	β5	0.60	0.51			0.45	NP	NP	NP	NP	NP

K410	G373	β5		1.36		0.64	0.38		1.09	0.49	0.64	0.38
I412	N371	β5		0.31	0.34	0.37	0.48					
$D336^{sc}$	$^{3}JNC\gamma$[c]	flat[d]	*1.43*	*1.31*	*1.30*	*1.23*	*1.24*	*1.43*	*1.31*	*1.30*	*1.37*	
$N339^{sc}$	$^{3}JNC\gamma$	flat	*2.48*	*2.37*	*2.50*	*2.60*	*2.42*					
$N350^{sc}$	$^{3}JNC\gamma$	dec	*2.89*	*2.61*	*2.33*	*2.48*	*2.40*	*2.49*	*2.31*	*2.42*	*2.42*	*1.82*
$D369^{sc}$	$^{3}JNC\gamma$	flat	*3.32*	*3.38*		*3.34*	*3.36*					
$N377^{sc}$	$^{3}JNC\gamma$	flat	*0.37*	*0.29*	*0.37*			*NP*	*NP*	*NP*	*NP*	*NP*
$D396^{sc}$	$^{3}JNC\gamma$	dec	*4.23*	*2.66*	*0.99*	*0.93*	*0.69*					
$N401^{sc}$	$^{3}JNC\gamma$	dec	*1.36*	*1.43*	*1.20*	*1.34*	*1.19*					

[a]Absolute values in Hz are given for all $^{h3}J_{NC'}$ and $^{3}J_{NC\gamma}$ couplings.

[b]NP – not present. Three resolved hydrogen bonds and one side-chain correlation are seen in the *cis* state but are missing in *trans* at all temperatures.

[c]The $^{3}JNC\gamma$ couplings are dpendent on the Asp/Asn sidechain χ_1 dihedral angle

[d]Temperature dependence of the $^{3}JNC\gamma$ couplings

Table S5. ^{3h}JNC' and ^{3}JNCγ couplings (Hz) in CUS-3i and P22i at various temperatures[a]

CUS3i							P22i								
**Don.**	**Acc.**	**274 ºK**	**286 ºK**	**295 ºK**	**298 ºK**	**305 ºK**	**Don.**	**Acc.**	**274 ºK**	**282 ºK**	**290 ºK**	**298 ºK**	**307 ºK**	**314 ºK**	**2º**
V230	A330	0.86	0.74	0.65	0.73	0.68									β1
S231	T256	0.67	0.14	0.49	0.87	0.61									β1
							Q234	L334	0.66	0.75	0.46	0.60	0.47	0.49	β1
H236	I306	0.75		0.68		0.21									β1
D242	M246		0.62	0.65	0.58	0.37									β1
G245	D242	0.39	0.29	0.36		0.27									turn
V248	A240	0.78	0.47	0.22	0.27	0.17									β2
G253	I302			0.83											β2
I255	V300	0.41	0.69	0.76		0.77	V259	V306	0.90	0.81	0.69	0.70	0.67	0.66	β2
V257	Q298	0.97	0.53	0.36	0.26	0.22	L261	T304	0.56	0.55	0.59	0.50	0.58	0.58	β2
S258	T229	0.96	0.63	0.75		0.62	S262	T229		0.85	0.91	0.77	0.81	0.78	β2
							T264	G303	1.17	1.45	1.24	1.16	1.48	1.36	irreg
G261	A259	1.00	0.96	0.85		0.63									irreg
G265	V292	0.76	0.43	0.82	0.53	0.63									β3
D266	Q263		0.21	0.49	0.45	0.17									β3
F268	F290	0.88		0.55	0.72	0.55	I273	F295			0.70	0.46	0.60	0.61	β3
A271	A331	0.72	0.95	1.2	1.0	1.0									β3
V273	I270	0.72	0.31	0.32		0.07									βi
S275	Q286	0.58	0.63	0.69	0.57	0.65									βi
H277	D282	0.34	0.19	0.77		0.74									βi
							A293	V278		0.42	0.31	0.31	0.35	0.30	irreg
F290	F268	0.87	0.99	0.74		0.94	F295	I273		0.36		0.71	0.66	0.76	β4
R291	S303	0.46	0.69	0.75		1.14	S296	T309		3.2	0.68	0.60	0.87	0.81	β4
							V297	D271		1.06	1.00		0.64	0.54	β4
L293	T301	0.68	0.75	0.99	0.99	1.22									β4
V300	I255	0.50	0.86	0.70		0.90	V306	V259		0.76	0.61		0.37	0.49	β5

T301	A294	0.62	0.74	0.74		0.80	E307	R299		0.83	0.59	0.58	0.64	0.57	β5
I302	G253	0.56	0.81	0.93	0.98	0.98	I308	A257			0.99	0.69	0.78	0.61	β5
S303	R291	0.76		0.83		1.0									β5
V309	V322	0.79	0.75	0.26	0.42	0.34									βii
N321	L307	1.10			0.75	0.93									βiii
D323	N274	0.19	0.27	0.38		0.49									βiii
							L334	E234			0.28	0.16	0.30	0.20	β6
							D336	A233	1.02	1.22	1.87	1.64	3.67	3.00	β6
							A337	A335		1.08	0.94		0.62	0.48	β6
I332	L228	0.56	0.45	0.42	0.41	0.34	V340	I228	0.67	0.40	0.70	0.34	0.42	0.37	β6
T333	T269		0.68	0.65	0.59	0.63	N341	S274		0.53	0.63	0.47	0.61	0.54	β6
L335	A267	0.51	0.54	0.66	0.72	0.67	L343	K272	0.80	0.63	0.71	0.50	0.52	0.51	β6
V276	*Q286^{sc}*			*0.91*											*bb-SC^{b}*
							N287	*Q283^{sc}*	*0.66*		*0.46*	*0.30*			*bb-SC*
							T309	*D253^{sc}*		*1.88*	*1.36*	*0.63*	*0.48*	*0.36*	*bb-SC*
							S319	*D317^{sc}*	*0.96*	*0.38*	*0.46*	*0.31*	*0.38*	*0.27*	*bb-SC*
D242^{sc}	flat		*0.23*	*0.51*	*0.41*	*0.47*									$^3JNC\gamma$
							N248^{sc}	dec				*0.96*	*0.65*	*0.63*	$^3JNC\gamma$
N244^{sc}	N.D.			*0.53*	*0.71*										$^3JNC\gamma$
							N251^{sc}	flat		*1.01*	*1.03*	*0.94*	*1.0*		$^3JNC\gamma$
							D253^{sc}	flat	*1.36*	*1.64*	*1.98*	*1.36*	*1.60*	*1.05*	$^3JNC\gamma$
D282^{sc}	flat	*0.52*	*0.49*	*0.48*	*0.50*	*0.65*									$^3JNC\gamma$
							D317^{sc}	dec	*3.45*	*3.23*	*3.24*	*2.63*			$^3JNC\gamma$
D313^{sc}	flat	*3.6*	*4.3*	*3.4*	*3.7*	*4.2*									$^3JNC\gamma$
							N341^{sc}	flat			*0.91*	*0.75*	*0.94*		$^3JNC\gamma$
							N344^{sc}	flat	*0.51*	*0.60*	*0.64*	*0.58*	*0.67*	*0.49*	$^3JNC\gamma$

[a]All of the reported couplings are absolute values in Hz. Rows in blue denote structurally equivalent H-bonds in the homologous P22i and CUS3i proteins

[b]H-bonds between backbone and sidechains (sc).

Table S6. Temperature dependence of mainchain H-bonds in HlyIIC.

			Major cis form or unresolved								Minor trans form							
Don.	Acc.	2°	HN (ppm)	N (ppm)	C' (ppm)	d_{NO}[a] (Å)	Slope[b] (Å/K)	SEM	R[c]	type[d]	HN (ppm)	N (ppm)	C' (ppm)	d_{NO}[a] (Å)	slope[b]	SEM (Å/K)	R[c]	type[d]
I331	Q327	α1	8.01	121.67	176.62	ND	ND	ND	ND	ND								
N332	N329	α1	8.47	117.90	178.33	3.04	0.0099	0.0017	0.97	inc	8.45	118.00	178.33	3.04	0.0063	0.0027	0.84	inc
L338[f]	V334	α1	7.79	115.70	178.57	ND	0.0050	0.0132	0.35	flat								
G343	G364	turn	8.45	111.96	174.90	2.83	-0.0062	0.0044	0.63	dec	8.41	111.96	174.62	2.84	-0.0131	0.0096	0.62	dec
S346	T357	β1	8.50	118.60	172.49	2.92	0.0021	0.0005	0.92	inc								
S348	K355	β1	9.03	114.12	174.24	2.90	-0.0011	0.0016	0.36	flat	8.99	114.12	173.93	2.90	-0.0025	0.0025	0.51	flat
N350	Q353	β1	9.21	123.80	174.77	2.88	0.0006	0.0023	0.19	flat	9.15	123.80	174.56	2.73	-0.0061	0.0029	0.77	dec
Q353	N350	turn	7.82	118.92	175.76	3.13	0.0096	0.0045	0.78	inc								
K355	S348	β2	9.24	128.52	171.90	2.82	-0.0003	0.0018	0.11	flat	9.27	128.52	171.88	2.75	-0.0020	0.0026	0.40	flat
T357	S346	β2	8.88	115.25	174.12	2.98	0.0007	0.0043	0.10	flat	8.88	115.25	174.12	2.98	0.0051	0.0018	0.85	inc
S359	K344	β2	8.59	113.00	173.59	3.10	0.0019	0.0043	0.25	flat								
I365	E386	irreg	8.31	121.33	176.46	2.82	-0.0229	0.0116	0.75	dec								
I365	K387	irreg	8.31	121.33	179.97	2.79	0.0008	0.0020	0.21	flat								
W372	F384	β3	9.23	121.64	171.18	2.80	0.0012	0.0014	0.44	flat								
G373	K410	β3	9.57	106.62	174.05	2.79	0.0022	0.0012	0.74	inc								
I374	Y382	β3	8.69	120.19	172.69	2.74	-0.0028	0.0008	0.92	dec	8.73	120.19	172.68	2.82	-0.0018	0.0002	0.98	dec
N377	Y406	irreg	9.70	127.89	174.06	3.01	0.0070	0.0023	0.86	inc	NP[e]	NP	NP	NP	NP	NP	NP	NP
V381	I374	β4	8.98	122.35	174.34	2.86	0.0024	0.0007	0.89	inc	8.90	122.35	174.18	2.82	0.0002	0.0034	0.03	flat
F384	W372	β4	8.87	116.72	174.72	2.86	-0.0028	0.0009	0.91	dec								
I393	T389	α2	7.80	119.16	177.63	3.01	0.0057	0.0007	0.98	inc								
S394	V390	α2	8.34	111.48	178.55	2.88	-0.0032	0.0012	0.82	dec								
D396	N392	α2	8.06	119.55	177.04	2.92	-0.0090	0.0020	0.93	dec								
I397	I393	α2	8.00	118.33	177.99	2.92	0.0017	0.0018	0.48	flat	8.05	118.33	178.17	3.14	0.0088	0.0028	0.87	inc
N398	S394	α2	8.12	117.24	175.86	3.00	0.0043	0.0018	0.82	inc								
G404	N352	irreg	8.52	107.11	173.64	2.91	0.0029	0.0011	0.82	inc	NP	NP	NP	NP	NP	NP	NP	NP
E408	F375	β5	9.29	125.20	175.43	2.95	0.0029	0.0012	0.93	inc	NP	NP	NP	NP	NP	NP	NP	NP
K410	G373	β5	9.15	122.00	171.61	3.00	0.0186	0.0012	0.99	inc	9.15	122.00	171.61	3.00	0.0124	0.0063	0.81	inc
I412	N371	β5	7.81	125.05	173.69	2.93	-0.0062	0.0012	0.96	dec								

[a]Heavy atom H-bond distances (d_{NO}) at a fixed temperature of 307 °K were calculated from $^{h3}J_{NC'}$ couplings using the empirical formula (Eq. 2) $d_{NO} = 2.75 – 0.25 \ln|^{3h}J_{NC'}|$

[b]Slopes calculated as $\delta\ln(d_{NO})/\delta T$. SEMs are the standard error of the means from linear regression.

[c]R-value for linear fiting of the $\ln(^{h3}J_{NC'})$ vs T data

[d]Type of temperature dependence for the H-Bond: increase, decrease, or flat. Flat dependences were those where the slopes were zero within experimental uncertainty or R < 0.6.

[e]NP – not present. Three resolved hydrogen bonds are seen in the *cis* form but are missing in the trans form at all temperatures. ND – not determined.

Table S7 – Temperature dependence of mainchain H-bonds in CUS-3i and P22i[a]

CUS3iD						P22iD						
Don.	Acc.	Slope[b] (Å/K)	SEM	R[c]	type[d]	Don.	Acc.	Slope[b] (Å/K)	SEM	R[c]	type[d]	2°
V230	A330	0.0019	0.0007	0.84	inc							β1
S231	T256	0.0035	0.0083	0.24	flat							β1
						Q234	L334	0.0023	0.0012	0.70	inc	β1
H236	I306	0.0089	0.0068	0.79	inc							β1
D242	M246	0.0065	0.0036	0.79	inc							β1
G245	D242	0.0023	0.0017	0.69	inc							turn
V248	A240	0.0125	0.0016	0.98	inc							β2
G253	I302	ND[e]	ND	ND	ND							β2
I255	V300	-0.0050	0.0019	0.89	dec	V259	V306	0.0019	0.0004	0.91	inc	β2
V257	Q298	0.0123	0.0009	0.99	inc	L261	T304	-0.0002	0.0005	0.16	flat	β2
S258	T229	0.0033	0.0009	0.76	inc	S262	T229	0.0009	0.0006	0.66	inc	β2
						T264	G303	-0.0007	0.0008	0.38	flat	irreg
G261	A259	0.0036	0.0012	0.92	inc							irreg
G265	V292	0.0006	0.0032	0.11	flat							β3
D266	Q263	0.0016	0.0012	0.09	flat							β3
F268	F290	0.0036	0.0017	0.83	inc	I273	F295	0.0004	0.0029	0.11	flat	β3
A271	A331	-0.0029	0.0014	0.75	dec							β3
V273	I270	0.0171	0.0049	0.93	inc							βi
S275	Q286	-0.0007	0.0009	0.40	flat							βi
H277	D282	-0.0087	0.0066	0.68	dec							βi
						A293	V278	0.0017	0.0012	0.61	inc	irreg
F290	F268	0.0001	0.0017	0.03	flat	F295	I273	-0.0056	0.0019	0.90	dec	β4
R291	S303	-0.0069	0.0010	0.98	dec	S296	T309	0.0077	0.0062	0.59	inc	β4
						V297	D271	0.0055	0.0006	0.99	inc	β4
L293	T301	-0.0048	0.0007	0.97	dec							β4

V300	I255	-0.0040	0.0022	0.79	dec	V306	V259	0.0043	0.0020	0.84	inc	β5
T301	A294	-0.0019	0.0005	0.94	dec	E307	R299	0.0020	0.0013	0.67	inc	β5
I302	G253	-0.0047	0.0009	0.95	dec	I308	A257	0.0040	0.0020	0.82	inc	β5
S303	R291	-0.0020	0.0009	0.92	dec							β5
V309	V322	0.0082	0.0035	0.81	inc							βii
N321	L307	0.0020	0.0021	0.69	flat							βiii
D323	N274	-0.0078	0.0004	1.00	dec							βiii
						L334	E234	0.0010	0.0005	0.14	flat	β6
						D336	A233	-0.0076	0.0016	0.93	dec	β6
						A337	A335	0.0063	0.0005	0.99	inc	β6
I332	L228	0.0037	0.0004	0.98	inc	V340	I228	0.0031	0.0020	0.61	inc	β6
T333	T269	0.0012	0.0010	0.66	inc	N341	S274	0.0000	0.0013	0.02	flat	β6
L335	A267	-0.0028	0.0008	0.90	dec	L343	K272	0.0028	0.0008	0.86	inc	β6

[a]H-bonds are listed as the NH donor (Don.) and O=C acceptor (Acc.). Rows in blue denote structurally equivalent H-bonds in the homologous P22i and CUS3i proteins

[b]Slopes calculated as $\delta\ln(d_{NO})/\delta T$. SEMs are the standard error of the means from linear regression.

[c]R-value for the linear fit of the data to $\ln(^{h3}J_{NC'})$ vs T data

[d]Type of temperature dependence for the H-Bond: increase, decrease, or flat. Flat dependences were those where the slopes were zero within experimental uncertainty or R < 0.6.

[e]ND – not determined.

Table S8 – Comparison between CUS-3i NMR and cryoEM H-bonds.

Donor NH	Acceptor O	dNO NMR (Å)	dNO cryo(Å)	<DHA cryo (º)[a]	exceptions / comments
G223	A336	Not seen	3.01	143.4	N-terminus, dynamic
L228	E226	Not seen	2.97	136.4	Bad H-bond angle, turn
V230	A330	2.86	2.88	161.0	**Agree**
S231	T256	2.92	2.90	165.6	**Agree**
H236	I306	2.85	2.97	169.0	**Agree**
D242	M246	2.86	2.90	164.5	**Agree**
G245	D242	3.00	2.99	155.5	**Agree**
V248	A240	3.13	3.09	163.6	**Agree**
N250	V248	Not seen	3.18	107.2	Bad H-bond angle, turn
R251	D249	Not seen	3.18	103.3	Bad H-bond angle, turn
Q252	D249	Not seen	3.48	130.5	Bad H-bond angle, turn
G253	I302	2.80	3.02	151.9	**Agree** (but distance is off)
I255	V300	2.82	2.85	170.0	**Agree**
V257	Q298	3.00	2.83	167.4	**Agree**
S258	T229	2.82	2.90	166.2	**Agree**
G261	A259	2.79	Not seen		**Disagree (missing cryoEM)**
G265	V292	2.80	2.93	177.3	**Agree**
D266	Q263	2.93	3.04	162.0	**Agree**
F268	F290	3.03	2.92	165.0	**Agree**
T269	T333	2.94	2.91	152.7	**Agree**
A271	A331	2.84	3.17	117.5	**Agree** (but distance is off)
V273	I270	3.03	3.45	174.7	**Agree** (but distance is off)
S275	Q286	2.84	3.07	163.2	**Agree** (but distance is off)
V276	N321	Not seen	3.18	139.0	Bad H-bond angle
H277	D282	2.81	2.83	162.8	**Agree**
K281	H277	Not seen	2.96	131.0	Bad H-bond angle, turn
T284	S275	Not seen	3.06	148.4	Spectral overlap precludes NMR
Q288	V273	Not seen	3.02	173.0	**Disagree (missing NMR)**

F290	F268	2.82	2.78	174.5	**Agree**
R291	S303	2.82	**Not seen**		**Disagree (missing cryoEM)**
V292	D266	**Not seen**	3.08	161.3	Spectral overlap precludes NMR
L293	T301	2.75	2.80	165.6	**Agree**
A296	T299	**Not seen**	2.82	163.5	Not seen in NMR, turn
T299	S296	2.89	2.89	159.4	**Agree**
V300	I255	2.84	2.86	174.0	**Agree**
T301	A294	2.82	2.92	175.5	**Agree**
I302	G253	2.77	2.80	168.8	**Agree**
S303	R291	2.80	2.88	159.3	**Agree**
V309	V322	3.08	3.13	137.8	**Agree**
N311	P308	**Not seen**	3.10	136.2	Bad H-bond angle, turn
S316	D313	**Not seen**	3.26	131.1	Bad H-bond angle, turn
R317	R314	**Not seen**	3.22	148.3	Not seen in NMR, turn
A320	R317	**Not seen**	3.43	162.5	Not seen in NMR, turn
N321	L307	3.02	2.94	123.3	**Agree**
D323	N274	2.83	2.89	152.1	**Agree**
I332	L228	2.92	2.96	158.9	**Agree**
T333	T269	2.89	2.94	175.0	**Agree**
L335	A267	2.85	2.74	153.2	**Agree**
M246	D242 Cγ (SC)	**Not seen**	2.96	157.6	Not seen in NMR, turn
Q263	D266 Cγ (SC)	3.29	2.84	161.0	**Agree** (but distance is off)
A267	N366 Cγ (SC)	**Not seen**	2.95	168.2	C-terminus, dynamic
N274	D323 Cγ (SC)	**Not seen**	2.85	159.4	Not seen NMR, side-chain
V276	Q286 Cδ (SC)	2.77	**Not seen**		Not seen cryo, side-chain
L307	N321 Cγ (SC)	**Not seen**	3.21	152.0	Not seen NMR, side-chain
T312	E325 Cδ (SC)	**Not seen**	2.90	155.6	Not seen NMR, side-chain
A315	D313 Cγ (SC)	**Not seen**	3.28	108.8	Spectral overlap precludes NMR

[a] <DHA is angle between the three atoms donor-H-acceptor in an H-bond.

Table S9 – Comparison between P22i NMR and cryoEM H-bonds[a]

Donor NH	Acceptor O	dNO NMR (Å)	5UU5 dNO[b] (Å)	<DHA (º)	8I1V dNO (Å)	<DHA (º)	exceptions / comments
V230	M338	2.84	3.15	156.2	2.99	154.9	Agree (but distance is off)
S231	T260	3.12	3.06	146.4	3.05	175.7	Agree
Q234	L334	2.94	3.44	123.5	Not seen		Agree (but distance is off)
F236	A314	2.81	Not seen		Not seen		Disagree
V242	E250	Not seen	2.98	145.2	2.80	161.3	Dynamic D-loop in solution, capsomer contact cryo-EM
G247	D244	Not seen	3.17	126.8	2.99	167.3	Dynamic D-loop in solution, capsomer contact cryo-EM
V250	Q242	Not seen	3.05	137.9	2.86	168.8	Dynamic D-loop in solution, capsomer contact cryo-EM
V252	A240	Not seen	3.02	158.1	Not seen		Dynamic D-loop in solution, capsomer contact cryo-EM
A257	E308	3.00	3.14	136.3	3.26	161.6	Agree
V259	V306	2.85	2.58	150.4	2.90	150.2	Agree
L261	T304	2.89	3.50	143.2	2.54	153.0	Agree (but distance is off)
S262	T229	2.80	2.56	164.7	2.65	148.2	Agree
T264	G303	2.65	3.31	142.2	2.81	162.7	Agree
G270	V297	Not seen	2.74	159.9	Not seen		Spectral overlap precludes NMR
D271	K268	Not seen	3.43	171.7	Not seen		Not seen in NMR, turn
I273	F295	2.88	2.73	159.6	2.59	156.3	Agree
A276	A339	3.09	3.22	162.3	2.91	155.3	Agree
V278	F275	3.26	3.43	155.2	Not seen		Agree
F280	Q291	Not seen	3.13	154.5	3.09	166.8	Dynamic S-loop in solution
L281	N329	Not seen	3.26	135.2	Not seen		Dynamic S-loop in solution
K286	E282	Not seen	2.59	139.0	2.79	146.0	Dynamic S-loop in solution
L289	F280	Not seen	3.07	140.5	3.04	167.2	Dynamic S-loop in solution
A293	V278	3.01	3.60	156.5	2.96	159.8	Agree
F295	I273	2.85	2.68	168.2	2.47	169.2	Agree
V297	D271	2.86	2.91	144.8	3.00	152.0	Agree
V298	E307	Not seen	3.00	144.4	2.56	160.7	Disagree
V301	H305	3.11	Not seen		3.04	123.6	Agree

D302	T304	3.11	Not seen		3.36	167.9	Agree
V306	V259	3.00	2.69	157.9	3.06	166.8	Agree
E307	R299	2.86	3.27	155.0	2.96	153.3	Agree
I308	A257	2.81	2.98	155.2	3.01	157.2	Agree
T309	S296	2.96	3.13	145.3	2.62	149.7	Agree
V313	K311	Not seen	2.82	127.0	Not seen		Bad H-bond angle, turn
L315	V330	3.01	2.71	124.9	3.15	158.0	Agree
R325	S321	Not seen	2.96	155.4	2.66	144.7	Disagree
A326	E323	Not seen	3.24	143.9	Not seen		turn
A327	E324	Not seen	3.21	157.9	3.16	162.2	turn
N329	V313	3.06	3.16	116.0	3.26	137.2	Agree
N331	K279	3.06	3.50	155.1	Not seen		Agree (but distance is off)
L334	E234	3.05	4.00	109.6	Not seen		Bad H-bond angle
D336	A233	2.42	Not seen		Not seen		Disagree
A337	A335	2.87	Not seen		Not seen		Disagree
V340	I228	2.97	3.04	130.9	3.33	161.3	Agree
N341	S274	2.87	2.84	172.3	Not seen		Agree
L343	K272	2.91	2.90	168.6	3.09	163.2	Agree
G232	D336 Cγ (SC)	3.27	2.78	132.1	Not seen		Backbone to sidechain
K268	D271 Cγ (SC)	Not seen	2.98	143.1	2.79	148.2	Backbone to sidechain
K279	N331 Cγ (SC)	Not seen	3.06	156.0	Not seen		Backbone to sidechain
N287	Q283 Cδ (SC)	2.91	Not seen		Not seen		Backbone to sidechain
T309	D253 Cγ (SC)	2.93	Not seen		Not seen		Backbone to sidechain
S319	D317 Cγ (SC)	2.99	Not seen		Not seen		Backbone to sidechain
S321	Q324 Cδ (SC)	Not seen	3.21	165.0	Not seen		Backbone to sidechain

[a] H-bonds were calculated using the server http://cib.cf.ocha.ac.jp/bitool/HBOND/

[b] PDB code 5UU5 and 8I1V are the 3.3 Å-resolution and 2.6 Å-resolution structures of phage P22, respectively.

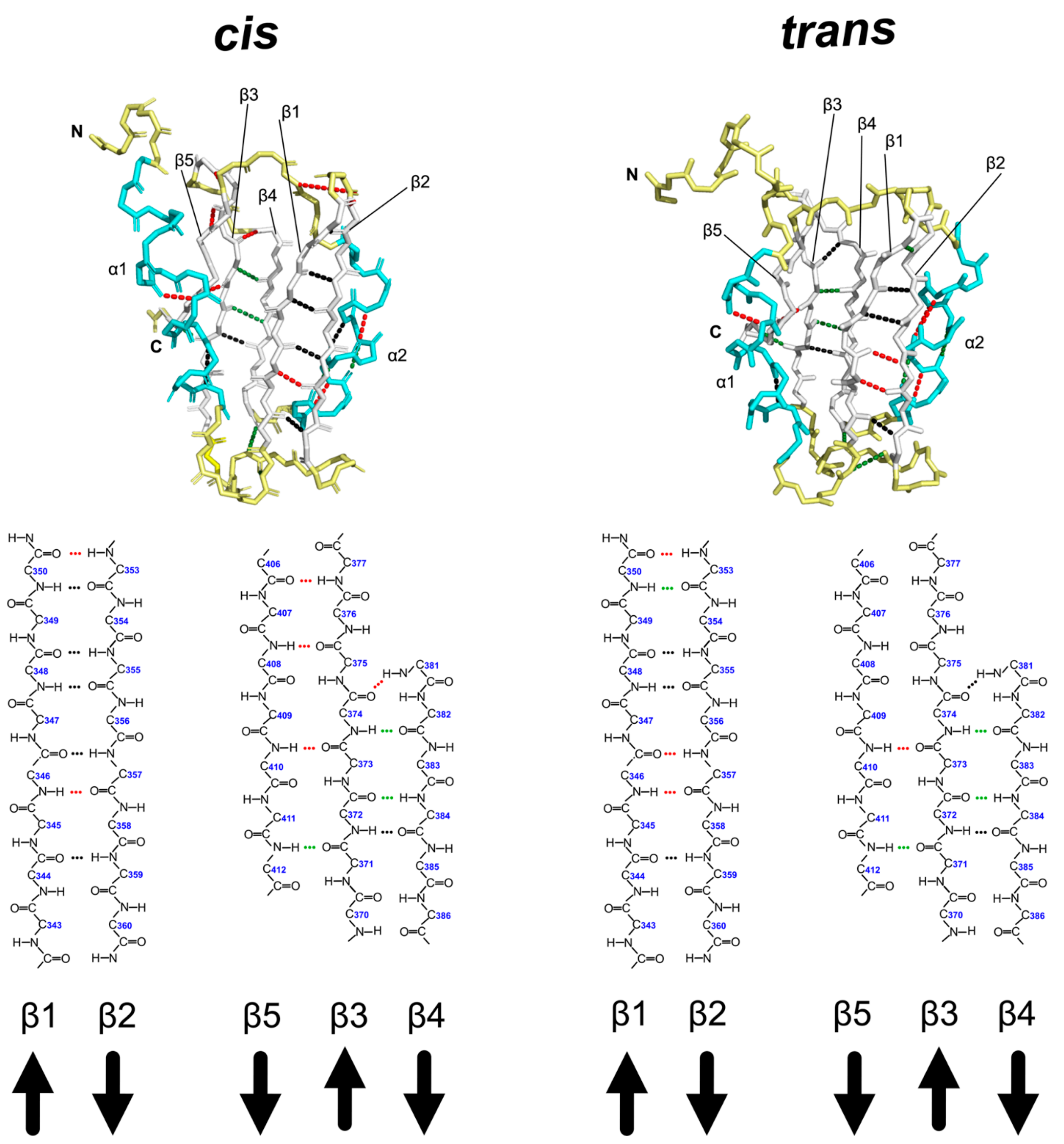

Supplementary Figure S1 Temperature dependence of β-sheet H-bonds in *cis* and *trans* HlyIIC. NMR structures closest to the ensemble averages for *cis* (PDB 6DZ5) and *trans* (PDB 6D53) HlyIIC are clored according to secondary structure: white – β-sheet, cyan – α-helix, yellow – irregular. For clarity, the β-sheets in the structures are illustrated schematically with H-bonds colored according to the effects of temperature on their lengths: red – increase, black – unchanged, green – decrease. Only H-bonds detected by lrHNCO experiments in this study are illustrated.

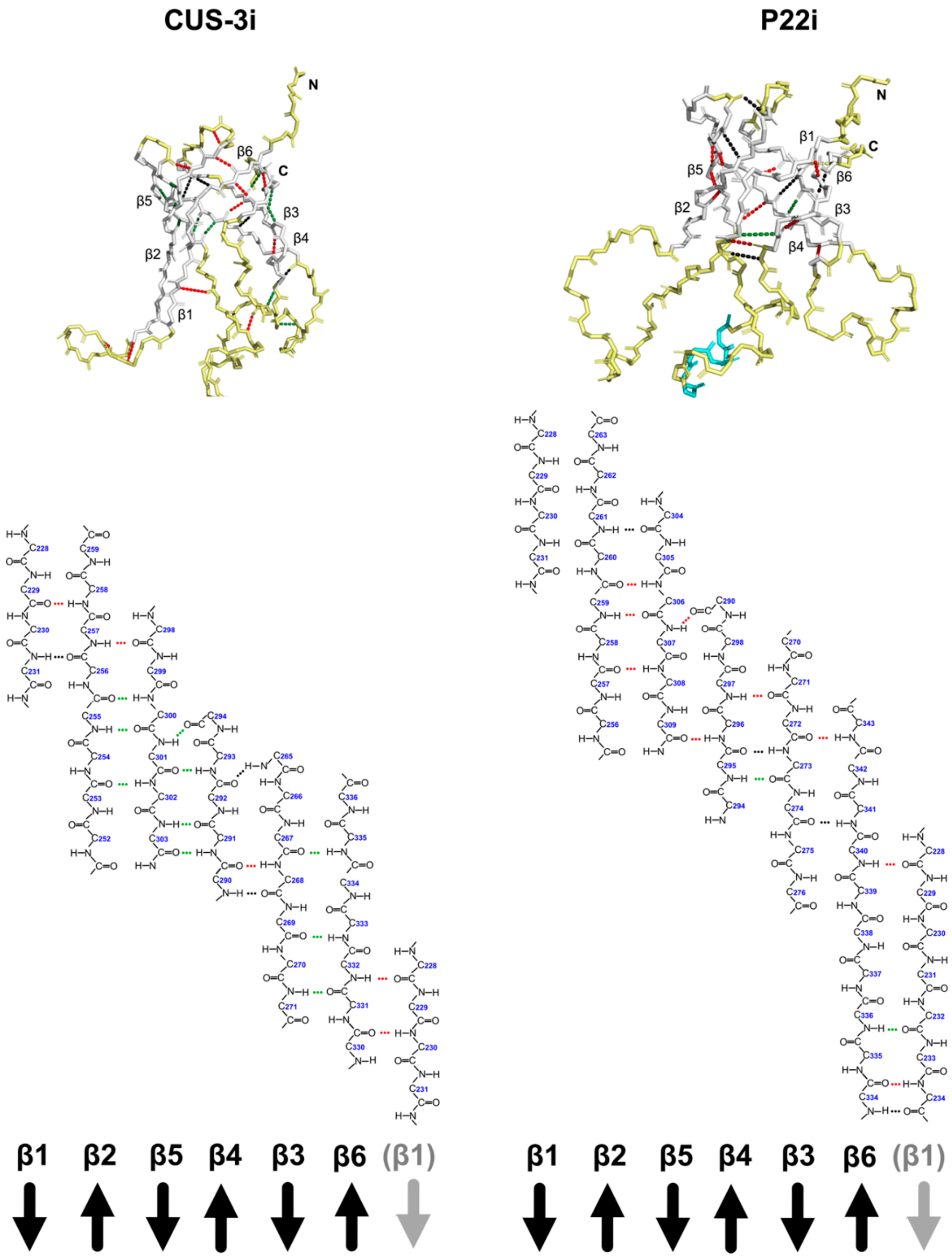

Supplementary Figure S2 Temperature dependence of β-sheet H-bonds in CUS-3i and P22i. NMR structures closest to the ensemble averages for CUS-3i (PDB 6MNT) and P22i (PDB 2M5S) are clored according to secondary structure: white – β-sheet, cyan – α-helix, yellow – irregular. For clarity, the β-sheets in the β-barrel structures of the two proteins are illustrated schematically with H-bonds colored according to the effects of temperature on their lengths: red – increase, black – unchanged, green – decrease. Only H-bonds detected by lrHNCO experiments in this study are illustrated. Strand β1 is shown twice for the β-barrel structures.
